# The effect of subliminal incentives on goal-directed eye movements

**DOI:** 10.1152/jn.00414.2021

**Published:** 2021-11-10

**Authors:** Vasko Kilian Hinze, Ozge Uslu, Jessica Emily Antono, Melanie Wilke, Arezoo Pooresmaeili

**Affiliations:** ^1^Perception and Cognition Lab, European Neuroscience Institute Göttingen: A Joint Initiative of the University Medical Center Göttingen and the Max-Planck-Society, Göttingen, Germany; ^2^Department of Cognitive Neurology, University Medicine Göttingen, Göttingen, Germany; ^3^German Primate Center, DPZ, Leibniz Institute for Primate Research, Göttingen, Germany; ^4^Leibniz ScienceCampus, Primate Cognition, Göttingen, Germany

**Keywords:** conscious awareness, reward, saccade, subliminal

## Abstract

Over the last decades, several studies have demonstrated that conscious and unconscious reward incentives both affect performance in physical and cognitive tasks, suggesting that goal pursuit can arise from an unconscious will. Whether the planning of goal-directed saccadic eye movements during an effortful task can also be affected by subliminal reward cues has not been systematically investigated. We employed a novel task where participants made several eye movements back and forth between a fixation point and a number of peripheral targets. The total number of targets visited by the eyes in a fixed amount of time determined participants’ monetary gain. The magnitude of the reward at stake was briefly shown at the beginning of each trial and masked by pattern images superimposed in time so that at shorter display durations participants perceived reward incentives subliminally. We found a main effect of reward across all display durations as higher reward enhanced participants’ oculomotor effort measured as the frequency and peak velocity of saccades. This effect was strongest for consciously perceived rewards but also occurred when rewards were subliminally perceived. Although we did not find a statistically significant dissociation between the reward-related modulation of different saccadic parameters, across two experiments the most robust effect of subliminal rewards was observed for the modulation of the saccadic frequency but not the peak velocity. These results suggest that multiple indices of oculomotor effort can be incentivized by subliminal rewards and that saccadic frequency may provide the most sensitive indicator of subliminal incentivization of eye movements.

**NEW & NOTEWORTHY** Reward incentives motivate humans to exert more effort, and they do so even when rewards are subconsciously perceived. It has been unknown whether these effects also extend to eye movements that have lower energetic demands compared with other movement types. We devised a behavioral task that required fast execution of multiple eye movements. Subliminal rewards enhanced the frequency and peak velocity of saccadic eye movements, with the most reliable effect observed for saccadic frequency.

## Graphical abstract

**Figure:**
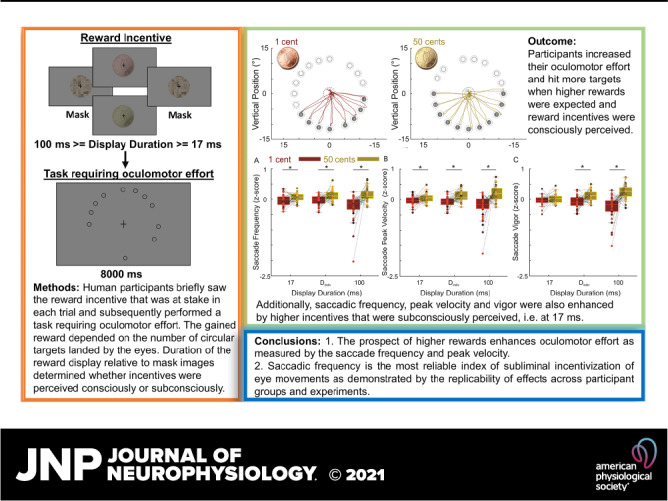

## INTRODUCTION

Human behavior is guided by a drive to maximize survival chances through avoiding unwanted situations and approaching desirable outcomes, that is, rewards (1). However, humans’ cognitive or physical resources, for instance, the extent to which they can exert physical effort in a task in order to obtain a reward, are limited. Hence, to efficiently interact with the surroundings, in a way that rewards are maximized while the effort to attain them is kept as low as possible, agents often set a certain goal at a given point in time and adjust their performance accordingly (2). A longstanding idea in cognitive neuroscience posited that the underlying processes of goal-pursuit arise from conscious awareness, where an agent is aware of the content of what one is experiencing or trying to achieve and the costs entailed. In the last decades, this idea has been challenged as researchers have repeatedly shown that goal-pursuit can also have its origin in an unconscious mind or even operate without conscious awareness (3–7). Here, we ask whether these effects also extend to goal-directed planning of eye movements.

Our study is inspired by the pioneering work of Pessiglione et al. (7) investigating whether goal-pursuit could be influenced by subliminally presented reward incentives. It was demonstrated that participants can adjust their level of physical effort in a handgrip force task dependent on the magnitude of the reward at stake in each trial. Remarkably, monetary reward incentives not only increased the exerted force when they were consciously perceived (i.e., were supraliminal) but also invigorated performance when presented below the conscious threshold (i.e., subliminally). The analysis of the simultaneously acquired fMRI data demonstrated that the same subcortical brain structures encode consciously as well as subconsciously perceived reward incentives. A number of subsequent studies replicated these results extending them to other behavioral paradigms requiring physical (8, 9) or mental effort (6, 10). Nevertheless, differences between the effects of conscious and unconscious reward incentives have also been reported. In a recent study, it was demonstrated that although both supraliminal and subliminal reward cues produce the same behavioral outcome, the underlying neural dynamics seem to be different (11). Furthermore, other studies showed that unconscious reward processing is rather limited when it comes to improving performance strategy and efficiency during complex task contexts (12, 13).

Reward effects on eye movements are ubiquitous, as reported by a number of previous studies (for a review see Ref. 14). For instance, neurophysiological studies in monkeys have demonstrated that the time to initiate a stimulus-driven saccade (i.e., saccade latency) is significantly shorter when a higher reward is expected (15–17). Furthermore, in rewarded conditions saccades typically have higher peak velocities (17, 18). Importantly, the reward-related increase in saccades’ peak velocity cannot be fully explained by the stereotypic relationship between saccades’ velocity and amplitude known as “main sequence” (19), demonstrating that reward incentives affect saccades’ velocity above and beyond biomechanical factors. Similar results were found in human psychophysics studies as monetary rewards were shown to increase saccade velocity (20) and vigor, that is, the peak velocity as a function of amplitude (21–23).

Despite the wealth of studies on the impact of rewards on different saccade parameters, it is not known whether humans can voluntarily control their saccadic “effort” based on the magnitude of the reward incentives (but see Ref. 24). Likewise, it is not known whether a putative reward-related adjustment of saccadic effort also occurs when the incentives are perceived subliminally, as has been the case for tasks requiring manual or cognitive effort (7, 10, 13). Examining the effects of subliminal incentives on eye movements not only allows to test the ubiquity of these effects across different effectors and task contexts but also helps to shed light on their underlying neural mechanisms. Planning of saccadic eye movements involves a well-defined neural machinery encompassing cortical and subcortical regions (25, 26), and reward effects have been reported at both levels (15, 27). Determining which aspects of saccadic effort can be influenced by subliminal rewards provides a first step toward delineating whether reward-driven modulation of oculomotor effort can be controlled through subcortical mechanisms as has been shown for other types of motor actions (7).

While studying the motivational effects of subliminal rewards, it is important to note that a task needs to be sufficiently demanding to warrant an adjustment of effort based on the expected rewards (28–30). Considering the frequency with which eye movements can be executed (i.e., 3 saccades/s), the energetic cost of moving the eyes is rather low (31). Therefore, an important question is whether an oculomotor task could be challenging enough to necessitate reward-based cost-benefit assessments and corresponding adjustment of effort. Here, we devised such a high-demand task, which required the rapid planning of a sequence of saccadic eye movements back and forth between a fixation point and several peripheral targets. The level of the reward at stake was briefly shown at the outset of each trial and varied in its visibility depending on its duration relative to two mask images.

We hypothesized that higher reward incentives enhance participants’ oculomotor effort irrespective of whether they are consciously perceived or not. Importantly, we tested this hypothesis while stringently controlling for the level of conscious awareness of the reward cues. The level of oculomotor effort was measured as the number of targets landed by the eyes (i.e., hit rates) as well as the frequency and peak velocity of all saccades. We found that the reward-induced enhancement of oculomotor effort was strongest when reward cues were fully visible but also occurred under conditions when the reward magnitude was perceived subliminally, especially in case of the saccade frequency.

## MATERIALS AND METHODS

In this study, we report the results of a main and a control experiment. The control experiment was conducted to test the reproducibility and robustness of our findings in the main experiment. We will explain the general methods used for the main experiment and point out the differences of the control experiment when they apply (see also the Supplemental Material; all Supplemental Material as well as the source data and analysis scripts are available at https://doi.org/10.17605/OSF.IO/BFSZW).

### Participants

Forty subjects (20 males and 20 females, age 19–45 yr; means ± SD age, 25.65 ± 5.04 yr) participated in the main experiment for financial compensation. All but six were right handed, had normal or corrected-to-normal vision, and were naïve to the hypothesis of the project. Two participants were excluded from the final analysis: for one participant part of the data were lost due to technical problems during the experiment and for the other participant more than 25% of all trials in the eye movement task had to be removed (see also under *Data Analysis*). Thus, the final sample comprised 38 subjects.

The sample size of the control experiment was based on the results of the main experiment in a group of participants who had reached subliminal perception at the shortest display duration [Cohen’s *d* (*d*_z_) = 0.65 for the effect of reward on saccades’ frequency, required number of participants, i.e., *n* for a power of 1–β = 0.8 in a one-tailed paired *t* test is *n* = 17] and was calculated in G*Power (32). A total of 17 participants were tested (8 males and 9 females, age 21–39 yr; means ± SD age, 25.59 ± 4.80 yr, all right handed and corrected-to-normal vision). The data of one participant were removed from further analysis, as calibration of the eye position was poor in some of the experimental blocks.

Before the experiment started and after all procedures were explained, participants gave their oral and written consent. The study was approved by the local ethics committee of the “Universitätsmedizin Göttingen” (UMG), under the proposal number 15/7/15.

### Stimulus Presentation and Eye Tracking Apparatus

Throughout the experiment, visual stimuli were displayed on a calibrated ASUS monitor subtending 1,280 × 800 pixels, and a refresh rate of 120 Hz placed at a distance of 60 cm to the participants. For tracking the eye position an EyeLink 1000 Plus system with a desktop mount was used (SR Research). The monocular eye position data (right eye) was acquired at a rate of 1,000 Hz for all but three participants. Due to a technical error, the data from these participants (*n* = 3) was recorded at a rate of 250 Hz and was interpolated to a sampling rate of 1,000 Hz during the offline analysis. All experiments were scripted in MATLAB, using Psychophysics toolbox (33). The EyeLink camera was controlled by the corresponding EyeLink toolbox in MATLAB (34). Before each block, the eye tracking system was calibrated to provide precise measurements using a 13-point standard EyeLink calibration procedure.

### Eye Movement Task

**Figure 1.**
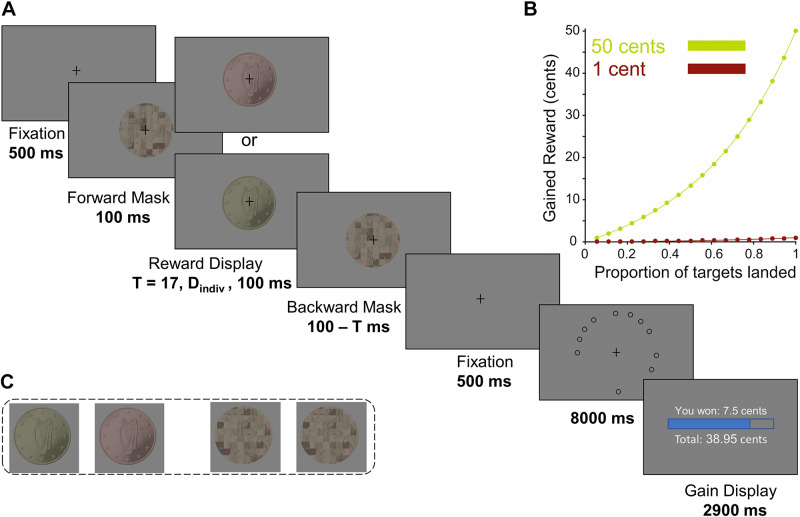
Experimental design. *A*: eye movement task. After an initial fixation period (500 ms), a sequence consisting of mask-reward-mask images was shown. The reward display was the image of either a 1 cent or a 50 cents coin, shown at one of three display durations: *T* = 17, D_indiv_, or 100 ms. D_indiv_ was an individual display duration (17 ms < D_indiv_ < 100 ms), set to the visibility threshold of each subject through a staircase procedure. After an additional fixation period (500 ms), 18 peripheral targets appeared on the screen at locations randomly chosen on each trial (eccentricity 11.5°). Participants had to look at as many circles as possible in a fixed time (8 s) and obtained a reward based on their performance at the end of the trial. *B*: the reward function. The gained reward was a fraction of the reward magnitude displayed at the outset of the trial (i.e. either 1 cent or 50 cents), scaled with the proportion of targets visited by the eyes using an exponential function (see Eq. *1* in the main text). *C*: high and low reward cues and mask images. From *left* to *right*: the 50 cents coin image (high reward cue), the 1 cent coin image (low reward cue), and the complementary checkerboard images that were used as forward and backward masks (see also the Supplemental Material).

We employed a speeded eye movement task with a 2 (reward: 50 cents versus 1 cent) by 3 (display duration: 17, D_indiv_ = an individual display duration, 100 ms) within-subjects factorial design. In this task, participants were exposed to the picture of a coin with either 50 cents (high reward) or 1 cent (low reward) value at the beginning of each trial (Fig. 1*A*). Participants could earn a fraction of the shown reward (Fig. 1*B*) by making a sequence of eye movements from the fixation point toward 18 peripheral target circles. In some trials, the coin image was displayed for 100 ms, allowing subjects to clearly discriminate the coin value, whereas in other trials, shorter durations of either 17 ms or an individual display duration (D_indiv_) were employed. The longest and shortest presentation durations (100 ms and 17 ms) were adapted from a previous study that used a similar masking procedure (7). The individual duration (D_indiv,_ where 17 ms < D_indiv_ < 100 ms) was determined through a staircase procedure for each participant. The staircase converged on the shortest display duration at which participants reported seeing the coin images (see the Supplemental Material). The reward display was superimposed in time by forward and backward pattern masks (Fig. 1*C*, see the Supplemental Material for details of the masking procedure). The eye movement task started with a practice block of 12 trials followed by five blocks of 42 trials each. This comprised 35 trials for each condition, counterbalanced for two values of the coin image (1 and 50 cents) presented at three display durations.

The sequence of events in a trial was as follows (Fig. 1*A*): a trial began with a fixation period (500 ms, fixation area 1°). Thereafter, a forward mask (100 ms) followed by the picture of a coin [either a 1 cent or a 50 cents Euro coin, displayed at 3 different durations (T): 17 ms, D_indiv_ or 100 ms] and a backward mask (100 ms minus coin display duration) were displayed while the participant was required to maintain fixation on the central-fixation cross overlaid on the picture of the masks and the coin. Subsequently, a second fixation period (500 ms) was followed by the main eye movement task. During the eye movement task, 18 circular targets (radius 0.75°, eccentricity 11.5°) surrounded a fixation cross at the center of the screen. In each trial, the position of the targets was selected by randomly drawing 18 samples out of 42 possible locations. This was done to discourage participants from planning their sequence of eye movements before the presentation of the targets, as target locations in each trial were unpredictable. Participants started the task by looking at the fixation cross and subsequently made an eye movement toward a target at any location. A target was considered to be “marked” (the term used for subjects; i.e., a hit) as soon as the eye position fell within a circular area with a radius of 1.2° from the center of the target. In our offline analysis, we counted a target-directed eye movement as a hit, if the eye position fell within an area twice as large as the threshold used online (radius = 2.4°, as for the data shown in Fig. 3*B*). Once a target was landed, it got filled with black color, thus indicating to the participant that they had successfully hit the target. A landed target remained black for the rest of the trial. To land on the next target, the participant had to first focus back on the fixation cross. The color of the fixation cross turned from black to red whenever the subject refocussed the fixation cross (within an area of 2.5° from the center of the fixation cross). This way, the participant received immediate feedback and was informed that they could continue to look at the next circle. As the participant moved their eyes away from the fixation cross, it turned black again. The total duration of the eye movement task was fixed to 8 s on each trial.

Participants’ goal in this task was to land on as many targets as possible in a fixed time interval (8 s). Depending on how many targets a subject could hit on each trial, they received a certain fraction of the coin’s value displayed at the outset, calculated as:

(*1*) $$Gained\;Reward=Coin\;Value\;\times \frac{10\frac{LT}{NT}-(1-\frac{LT}{NT})}{10},$$

where *LT* is the number of landed targets, *NT* corresponds to the total number of targets, and Coin Value is either 1 cent or 50 cents (Fig. 1*B*). The exponential function was used to potentiate the effect of incentives, as participants could readily see that their number of hits had a strong influence on the gained rewards. The gained reward was visually displayed on the monitor at the last phase of each trial and stayed in view for 2,900 ms. Here, participants could see how much money they gained during the last trial and also the amount they had earned so far in the current block of the experiment (each block consisted of 42 trials). In addition, a reward bar was shown to illustrate the progress of the reward earning during a block of the experiment. This bar was scaled to the total maximum amount that a participant could earn during a block of the experiment.

In the control experiment, coin images were presented at either a long (100 ms) or a short (D_indiv_ ms) display duration. D_indiv_ was determined through a staircase procedure similar to the main experiment (see the Supplemental Material). Importantly, in addition to using an adaptive method to determine the D_indiv_, we further adjusted the luminance contrast of the coin images to ensure that all participants perceive the coin images at a truly subliminal level. The luminance contrast of coin images at 100 ms was set to 100%.

### Assessing Participants’ Level of Conscious Awareness of Reward Cues

We used an independent “4-alternative forced choice” (4AFC) task adapted from a previous study (7) to assess the subjective visibility of the reward cues. The 4AFC task was structured in the same way as the eye movement task with the exception that after the second fixation period, a question with four alternative answers was displayed on the screen. Participants had to indicate which coin they saw or guessed to have seen by pressing one of the four designated keyboard keys (either the “A” or “S” key indicating “seen 50 cents” or “seen 1 cent”, or the “D” or “F” key indicating “guess 50 cents” or “guess 1 cent”, respectively). All conditions of this task were counterbalanced with 20 repetitions per incentive value and reward timing. The 4AFC task was repeated two times, once before and once after the eye movement task, and participants’ responses were pooled across the two repetitions.

### Data Analysis

**Figure 2.**
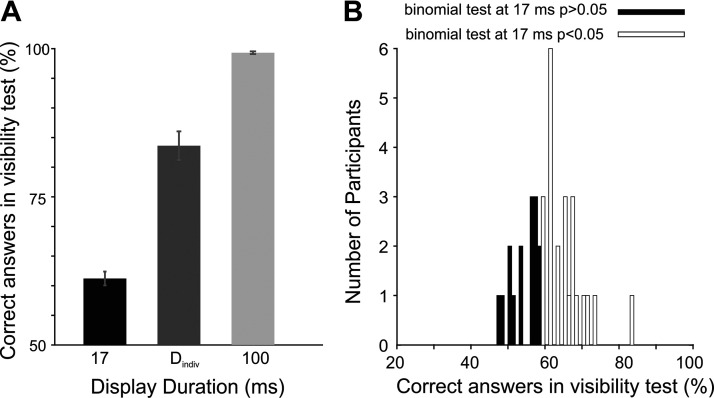
Results of the 4AFC visibility test. *A*: the subjective visibility of coin images at each display duration was assessed by measuring their percentage of correct discrimination in an independent 4AFC task. Correct answers were significantly above chance level at the two longer display durations but not at 17 ms, albeit in the latter case a trend was found (*P* = 0.07). *B*: the distribution of correct answers at 17 ms across participants. To control for the difference in the subjective visibility of coins across participants, the mean-centered probability of correct answers at 17 ms was included as a covariate in our analyses. In addition, we also tested the probability of correct answers against chance level in each participant using a binomial test and re-examined our effects while including participant group (marked in black or white) as a factor.

Participants’ subjective reports in the 4AFC task were analyzed following the method used by Pessiglione et al. (7) according to which subliminal perception needs to meet two criteria. First, correct answers (either “seen” or “guess”) should not differ from chance level. Second, the probability of the “seen” responses should not be different from 0. To test for these criteria, one-tailed one-sample *t* tests were used to test whether “correct” or “seen” answers at a group level were different from chance (50%) or 0, respectively. In addition, we also tested our effects with a more stringent criterion at the individual level, where the probability of correct answers in each participant was tested against chance level using a one-sided binomial test (implemented in MATLAB, chance level = 0.5, cut-off *P* for significance α = 0.05, Fig. 2*B*).

To assess different saccade parameters, eye position data of each trial from the beginning to the end of the eye movement task was analyzed (total duration 8,000 ms). Trials with >25% missing data in this period were discarded from the analysis. To detect saccades, eye position samples in a trial were smoothed using a Savitzky–Golay low-pass filter with an order of 2 and length of 20 ms (35). Saccade onsets were defined as the moment when a sample exceeded a two-dimensional velocity threshold of 35°/s. Saccade offsets were calculated as the first sample where the eye position velocity and acceleration dropped below 35°/s. In addition, saccades with an intersaccadic interval shorter than 40 ms and a duration shorter than 10 ms were discarded from the analysis to avoid contamination of the saccades by the eye-tracker noise. We also tested whether we obtain similar results when saccades were detected based on an adaptive algorithm as introduced by Nyström and Holmqvist (35). This algorithm can be applied to the data of individual trials where the threshold to detect saccades is set based on the noise-level of each trial in an adaptive manner, hence accounting for variability of noise level across trials, task conditions, and subjects. Furthermore, as the local noise level of samples is taken into account when the start and end of the saccade are estimated, the adaptive method is especially suitable for situations when participants scan the visual scene by making multiple eye movements, as is the case in the paradigm we employed here. We used the adaptive algorithm while setting the peak threshold equal to the mean plus 4 standard deviation of the trial velocity, saccade onset threshold equal to the mean plus 2.5 standard deviation of the trial’s velocity, and the minimum saccade duration at 10 ms. Saccades with preceding fixation durations shorter than 40 ms were discarded from the analysis (35). For both methods of saccade detection, the reported values of peak velocity and vigor were measured based on the maximum two-dimensional velocity of the samples between the onset and the offset of the saccade (i.e., the absolute maximum of the 2-D velocity samples—calculated from the Savitzky–Golay low-pass filtered data—between the onset and offset of the saccade). Saccade amplitude was calculated as the Euclidian distance between the eye position samples at the onset and offset of the saccade.

We computed a within-subject measure of saccade vigor, as shown in Fig. 4*C* using a method employed in a previous study (21). To this end, we measured the amplitude (represented by *x*) and the peak velocity (represented by *v*) of all saccades across all trials of each participant. Subsequently a hyperbolic function in the following form was fit to the saccade amplitude data:

(*2*) $$V_{n}=\alpha_{n}(1-\frac{1}{1+\beta_{n}x}),$$

where α̂*_n_* and β̂*_n_* characterize the hyperbolic relationship between the peak velocity and amplitude of the saccades. Based on these parameters, for each participant an expected saccade velocity given each saccade amplitude was calculated *v̂_n_*(*x*). Note that the fit parameters are computed from all saccades of a particular individual and hence the expected velocity solely reflects the stereotypical biomechanical relationship between saccades’ velocity and amplitude captured by the main sequence (19). Finally, the ratio between the measured and the expected average peak velocities was calculated for each condition (i.e., reward level and display duration). This ratio represents a within-subject measure of saccade vigor above and beyond the influence of saccade amplitude, with ratios >1 reflecting a greater than average vigor in a certain condition in an individual.

To quantify the saccade landing error, we first determined whether a saccade was directed toward the peripheral targets or the fixation point. Subsequently, we measured the 2-D Euclidian distance of the end point of the saccade from its respective destination as the saccadic landing error. Target-directed saccades were defined as saccades that the distance of their end point from any of the peripheral targets was smaller than their distance from the fixation point. The remaining saccades were categorized as fixation-directed.

All statistical analyses were done using the MATLAB Statistics Toolbox. For statistical inferences, we analyzed different indicators of the exerted effort (i.e., number of hits and frequency, peak velocity, and vigor of all saccades irrespective of their landing position) by carrying out a series of repeated measures ANOVAs (functions: fitrm, ranova in MATLAB). The ANOVAs included two within-subjects factors: reward (2 levels: 1 or 50 cent) and display duration (3 levels: *T* = 17 ms, D_indiv_, and 100 ms in the main experiment; or T = D_indiv_ and 100 ms in the control experiment) and one between-subject covariate (i.e., the mean-centered visibility scores at 17 ms as depicted in Fig. 2*B*) as independent factors. To test saccadic parameters with the same statistical model, raw values of saccadic frequency and peak velocity were *z*-scored across all trial of each individual and type of saccadic parameter was added as a within-subjects factor to the ANOVAs. In addition, we also tested our effects when participants were explicitly divided into two groups based on whether they could identify rewards significantly above the chance level or not and included participant group as a between-subject factor in our analyses. In all cases, a Greenhouse–Geisser correction was applied to the degrees of freedoms and *P* values of ANOVAs to account for violations of the sphericity assumption. Significant effects from the omnibus ANOVAs were further investigated by performing post hoc paired comparisons (using the function multcompare in MATLAB, with Bonferroni correction). Effect sizes in ANOVAs are reported as partial eta-squared ($\eta_{p}^{2}$) and in pairwise comparisons as Cohen’s *d* (i.e., *d*_z_: 36) throughout.

## RESULTS

### Results of the 4-AFC Visibility Test in the Main Experiment

To determine whether participants perceived the reward incentives above or below the threshold for subliminal perception, we first analyzed the data of the 4-AFC visibility test (Fig. 2, see materials and methods). This analysis showed that at the two longer display durations correct responses were significantly higher than the chance level (for *T* = 100 ms: means ± SD = 99.5 ± 0.92%, *t*_37_= 52.12, *P* < 10^−10^, *d*_z_ = 8.34; for *T* = D_indiv_: means ± SD = 84.6 ± 13.8%, *t*_37_= 1.47, *P* = 0.017, *d*_z_ = 0.4, one-tailed one-sample *t* test for a difference from chance level = 50%). At the shortest display duration (*T* = 17 ms) correct responses were not significantly different from chance, albeit a trend was found (mean = 61 ± 7%, *t*_37_= 1.47, *P* = 0.075, *d*_z_ = 0.24). Analysis of the “seen” responses showed that they were not significantly different from 0 at 17 ms (means ± SD = 10 ± 13%, *P* = 0.219), but they significantly differed from 0 at the other two display durations (*T* = D_indiv_: means ± SD = 44 ± 12% and *T* = 100 ms: 98 ± 01.9%, both *P*s < 10^−3^). These results indicate that the reward incentives were perceived consciously at 100 ms, as intended. However, the individual display duration set at a level where participants subjectively reported not seeing the coins (i.e., D_indiv_) proved to be above the level of conscious awareness when tested by a 4AFC task. At the shortest display duration (17 ms), subliminal perception was achieved according to one of the criteria (i.e., the “seen” responses) whereas the analysis of the correct responses showed a trend suggesting that for some participants this display duration has been longer than the threshold for subliminal perception (Fig. 2*B*). Therefore, to control for the differences in the level of conscious awareness of reward incentives across participants, each individual’s visibility score, i.e., the mean-centered proportion of correct responses at the shortest display duration, was included as a between-subjects covariate in all our subsequent analyses. In addition, we also tested the robustness of our effects by carrying out a second analysis where a more stringent criterion was used at the individual level to test whether the probability of correct responses in each participant was significantly higher than the chance level using a binomial test (Fig. 2*B*). Using this criterion, at 17 ms 15 participants had chance-level performance whereas 23 participants could identify coin images significantly above chance level (total sample size *n* = 38).

### Consciously Perceived Reward Incentives Increase the Number of Hits in a Goal-Directed Oculomotor Task

**Figure 3.**
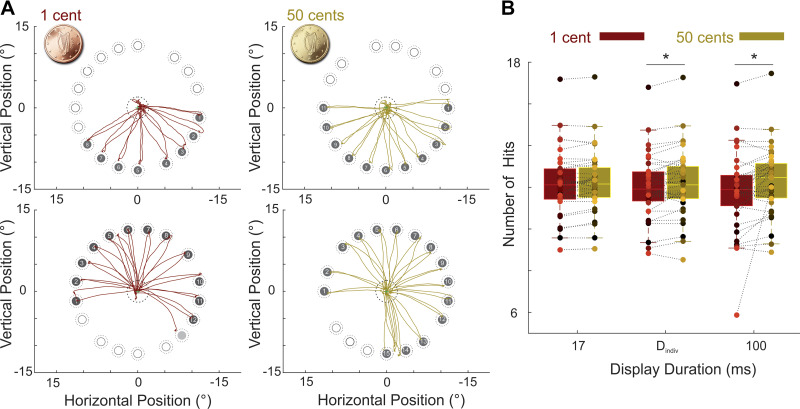
Performance in the eye movement task. *A*: eye position traces of example trials. *Top*: the data of one example participant in a trial with low (*left*, copper traces) and a trial with high (*right*, gold traces) reward is shown. The numbers inside each circular target indicate the order with which the participant looked at that location and the eye movement was counted as a hit. *Bottom*: same as the *top* panel for another example participant who had overall higher number of hits. The target filled with brighter gray color on the left side (low reward condition) was not detected as a hit online but was counted as a hit when a more liberal threshold was used offline. To account for such cases, we based all our analyses on the offline threshold (i.e. counting saccades landed < 2.4° from the target as a hit). *B*: hit rates. The total number of hits across all participants and all display durations for low (copper color) and high (gold color) reward values of the coins. Colored dots depict the data of individual participants. * *P* < 0.05.

To confirm that our eye movement task created a demanding situation where the exerted effort is adjusted based on the expected rewards, we first examined participants’ number of hits at the longest display duration where rewards were consciously perceived (100 ms). Figure 3*A* illustrates the eye position traces of two example subjects. For each subject, the data of two trials, one with a low and the other with a high reward cue (images of 1 cent and 50 cents coins, respectively) are shown. It can be readily seen that when the amount of expected reward was high, participants made more eye movements and hit more targets compared with when the reward at stake was low. The reward effect was observed both for participants who had overall lower number of hits (Fig. 3*A*, *top*), as well as those with higher hit rates (Fig. 3*A*, *bottom*, hit rates refer to saccadic eye movements that landed <2.4° from the center of a target, see also the materials and methods). To test the reward effect across participants and different display durations, a repeated-measures ANOVA was performed with reward (2 levels: high or low) and display duration (3 levels: 17, D_indiv_, and 100 ms) as the within-subjects factors and visibility scores at the shortest display duration (derived from the 4AFC task) as a between-subjects covariate (see also Table 1). This analysis revealed a main effect of reward as the number of hits was significantly higher for trials with high (50 cents) compared with low (1 cent) incentives [means ± SD = 12.23 ± 1.56 and 11.98 ± 1.61 for high compared with low reward incentives, respectively, *F*(1,36) = 10.98, *P* = 0.002, $\eta_{p}^{2}$= 0.23]. We also found a significant interaction effect between reward and display duration [*F*(1,36) = 4.15, *P* = 0.036, $\eta_{p}^{2}$= 0.10]. Other main and interaction effects did not reach statistical significance (all *P* values >0.1). Post hoc pairwise comparisons showed a significant effect of reward at the two longer display durations [for *T* = D_indiv_: *P* = 0.019 and Cohen’s *d* (*d*_z_) = 0.39; for *T* = 100 ms: *P* = 0.010 and *d*_z_ = 0.44) but the effect at 17 ms did not reach significance (*P* = 0.103, *d*_z_ = 0.26). These results indicate that participants amped up their effort and hit more targets when reward incentives were consciously perceived, whereas at the shortest display duration where the incentives were near subliminal threshold the effect was smaller and did not reach statistical significance.

**Table 1.** Overview of all performance measures analyzed in this study for the main and the control experiment

Main Experiment						
Reward and Duration	50 Cents, 17 ms	50 Cents, D_indiv_ ms	50 Cents, 100 ms	1 Cent, 17 ms	1 Cent, d_indiv_ ms	1 Cent, 100 ms
Number of hits	12.19 ± 1.56	12.20 ± 1.60	12.31 ± 1.57	12.11 ± 1.59	12.02 ± 1.52	11.81 ± 1.88
Saccade frequency	31.71 ± 3.28	31.88 ± 3.28	31.95 ± 3.31	31.44 ± 3.14	31.47 ± 3.32	30.73 ± 4.03
Peak velocity, °/s	387.54 ± 53.38	389.44 ± 54.79	391.62 ± 54.77	385.47 ± 53.59	383.89 ± 51.21	379.77 ± 52.37
Vigor	0.940 ± 0.03	0.944 ± 0.03	0.951 ± 0.03	0.937 ± 0.03	0.934 ± 0.02	0.926 ± 0.03
Amplitude, °	9.65 ± 0.74	9.65 ± 0.76	9.63 ± 0.74	9.60 ± 0.73	9.59 ± 0.69	9.55 ± 0.73
End point error, °	1.23 ± 0.28	1.23 ± 0.28	1.25 ± 0.28	1.22 ± 0.27	1.23 ± 0.28	1.22 ± 0.27

Control Experiment				
Reward and Duration	50 Cents, D_indiv_ ms	50 Cents, 100 ms	1 Cent, d_indiv_ ms	1 Cent, 100 ms
Number of hits	11.76 ± 1.70	11.76 ± 1.63	11.59 ± 1.52	11.56 ± 1.63
Saccade frequency	30.52 ± 3.29	30.39 ± 3.37	30.21 ± 3.17	30.19 ± 3.25
Peak velocity, °/s	364.43 ± 65.75	366.99 ± 66.08	363.27 ± 66.03	361.58 ± 65.33
Vigor	0.876 ± 0.04	0.878 ± 0.04	0.873 ± 0.04	0.870 ± 0.04
Amplitude, °	9.42 ± 0.67	9.52 ± 0.61	9.42 ± 0.61	9.40 ± 0.63
End point error, °	1.25 ± 0.21	1.26 ± 0.21	1.26 ± 0.21	1.25 ± 0.23

The number of hits and frequency, peak velocity, vigor, amplitude, and end point error of all saccades are shown for high (50 cents) and low (1 cent) reward incentives, displayed at different durations. Note that subminimal perception of rewards was observed for the display duration *T* = 17 ms in the main experiment and at *T* = D_indiv_ in the control experiment. Reported values are means ± SD. The number of participants was *n* = 38 in the main experiment and *n* = 16 in the control experiment.

### Incentive-Driven Modulation of Saccadic Frequency and Peak Velocity

**Figure 4.**
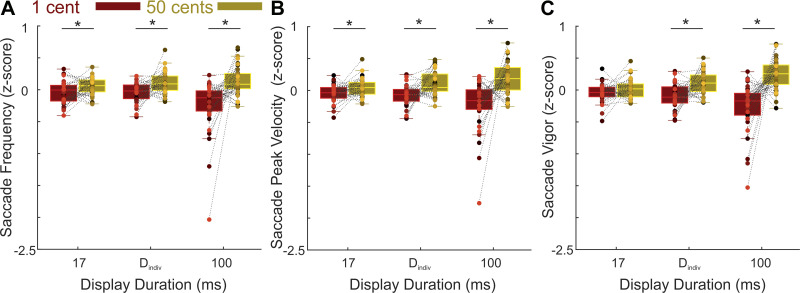
Influence of reward incentives on saccadic parameters. *A*: the effect of low (1 cent, copper color) and high (50 cents, gold color) reward incentives on the saccade frequency (i.e. the total number of saccades made in a trial irrespective of their landing position). *B*: same as *A* for the saccade peak velocity *C*: same as *A* for the saccade vigor. Colored dots depict the data of individual participants. *P < 0.05.

Hit rates in our task determined participants’ obtained reward and hence were the most direct measure of the effort expended in prospect of reward. However, hit rates pertain only to a subset of the saccades that landed adjacent to a target and disregard saccades that aimed to reach a target but failed to do so. To obtain a more sensitive estimation of the total amount of spent effort, we next inspected all saccadic eye movements made during a trial irrespective of their landing position. For these eye movements, we assessed the frequency (i.e., the total number of saccades made within the 8 s timeout duration of each trial) as well as the peak velocity/vigor of the saccades that are the major determinants of spent effort in our speeded eye movement task (Fig. 4 and Table 1). Increasing the frequency of saccades comprising either large saccades between the fixation point and the targets or smaller corrective saccades that bring the target or the fixation point to the center of the gaze, ensures that participants could hit more targets during a trial when higher rewards are at stake. Similarly, reward-based adjustment of saccades’ peak velocity, through increasing the velocity when expected reward is high and decreasing the velocity when rewards are low, ensures that the cost of making a fast saccade is optimally tuned to the expected reward. In order to test both saccadic parameters with the same statistical model, we *z*-transformed the raw values of each parameter across all trials of an individual (for an overview of raw value see Table 1).

We found that higher reward incentives increased the frequency of saccades across all display durations (means ± SD = 31.84 ± 3.27 and 31.21 ± 3.37 for high and low rewards, respectively, see Table 1). Similarly, across all display durations saccades had higher peak velocity when reward incentives were higher (means ± SD = 389.53 ± 51.96°/s) compared with when incentives were lower (means ± SD = 383.04 ± 54.17°/s). To test the reward effects on saccades’ frequency and peak velocity, we subjected both measures to an ANOVA (Fig. 4). The dependent factor of the ANOVA was the *z*-scored frequency and velocity of the saccades and the independent factors comprised three within-subjects factors (reward, display duration, and the type of the parameter: frequency vs. velocity) and one between-subjects covariate; i.e., the visibility scores at 17 ms. This analysis revealed a significant main effect of reward [*F*(1,36) = 26.13, *P* < 10^−4^, $\eta_{p}^{2}$= 0.42] and a reward × display duration interaction [*F*(2,72) = 10.60, *P* < 10^−3^, $\eta_{p}^{2}$= 0.23]. Post hoc pairwise comparisons revealed a significant effect of reward on the frequency (for *T* = 17 ms: *P* = 0.013 and *d*_z_ = 0.43; for *T* = D_indiv_: *P* = 0.005 and *d*_z_ = 0.50 and for *T* = 100 ms: *P* < 10^−3^ and *d*_z_ = 0.66) as well as the peak velocity of saccades (for *T* = 17 ms: *P* = 0.014 and *d*_z_ = 0.40; for *T* = D_indiv_: *P* = 0.001 and *d*_z_ = 0.63; for *T* = 100 ms: *P* < 10^−3^ and *d*_z_ =0.69) at all display durations. Therefore, the effect of reward incentives on saccadic parameters was stronger at the longer display durations but it also reached significance at 17 ms when the perception of reward magnitude was near the subliminal threshold. Importantly, the three-way interaction between reward × display duration × saccadic parameter did not reach significance [*F*(2,72) < 0.5, *P* > 0.1, $\eta_{p}^{2}$= 0.01], thus ruling out a dissociation between the reward-driven modulation of saccade frequency and peak velocity. Other main and interaction effects were nonsignificant (all *P* values >0.1). These results are in line with previous reports showing that reward incentives can boost the oculomotor effort even when they are perceived subliminally (7).

Saccadic peak velocity increases in a stereotyped manner when saccade amplitude increases, a relationship known as the main sequence (19). We therefore performed a follow-up analysis where we tried to remove the effect of saccade amplitude from peak velocities by calculating a within-subject estimate of saccade vigor (21). Analysis of saccadic vigor (*z*-scored) revealed a main effect of reward [*F*(1,36) = 29.72, *P* < 10^−5^, $\eta_{p}^{2}$= 0.45] and an interaction between reward and visibility level [*F*(2,72) = 16.98, *P* <10^−4^, $\eta_{p}^{2}$= 0.32]. Other main and interaction effects did not reach statistical significance (all *P* values >0.1). Follow-up, post hoc tests at each visibility level showed a significant effect of reward at the two longer display durations and a trend at 17 ms (for *T* = 17 ms: *P* = 0.059 and *d*_z_ = 0.32; for D_indiv_: *P* < 10^−3^ and *d*_z_ =0.64; for *T* = 100 ms: *P* < 10^−5^ and *d*_z_ = 0.87). Thus, the analysis of the saccadic vigor showed overall the same results as those observed for the peak velocity, with the exception that at 17 ms only a trend for a significant effect of reward was found.

Performance in the eye movement task depends on both speed (i.e., the frequency of all saccades and the velocity of each saccade) and accuracy of saccades in landing on the target. We next examined whether the saccadic error, i.e., 2-D Euclidian distance of the saccades’ end points from the targets, was changed with reward incentives at different visibility levels. An ANOVA with reward and display duration as within-subjects factors and visibility scores as between-subjects covariate did not reveal any significant main or interaction effect (all *P* values >0.05). Therefore, our results cannot be explained by a reduction of saccades’ accuracy.

To account for the fact that our sample of participants comprised individuals with different degrees of conscious awareness of incentives at the shortest display duration, we have thus far included participants’ visibility scores as a between-subjects covariate in all our analyses. We next tested whether the effect of subliminal incentives at the shortest display duration (17 ms) can also be observed in a group of participants where every subject had reached subliminal perception of reward magnitude at 17 ms. For this purpose, participants were divided into two groups based on whether the binomial probability of correct identification of coin value in the 4AFC task in each individual was significantly higher than the chance level or not (Fig. 2*B*, see materials and methods). In a group of participants where every individual had perceived rewards at chance level, the saccadic frequency was significantly enhanced by higher rewards (*P* = 0.03, *d*_z_ = 0.60) but there was almost no effect on the peak velocity (*P* = 0.69, *d*_z_ = −0.10). In participants who perceived rewards above the chance level however, there was a significant effect of reward on saccades’ peak velocity (*P* = 0.001, *d*_z_ = 0.82) but the effect on saccadic frequency was smaller and did not reach significance (*P* = 0.12, *d*_z_ = 0.34). Despite differences between groups, an ANOVA on saccadic parameters (*z*-scored frequency and peak velocity of saccades at 17 ms, while including participant group as a between-subjects factor) only revealed a significant main effect of reward [*F*(1,36) = 16.48, *P* < 10^−3^, $\eta_{p}^{2}$= 0.31], but the interaction of group with other factors did not reach significance [2-way reward × group interaction: *F*(1,36) = 3.07, *P* = 0.09, $\eta_{p}^{2}$= 0.08, and 3-way reward × group× saccade parameter interaction: F(1,36) = 3.04, *P* = 0.073, $\eta_{p}^{2}$= 0.08].

Taken together, we found a robust enhancement of the saccade frequency and velocity/vigor in prospect of higher rewards. These reward-driven modulations were strongest at the longer display durations, but also reached significance at *T* = 17 ms when perception was near-threshold. No statistically significant difference was found between the effect of reward on the frequency versus the peak velocity of saccades. In a group of participants where each individual had perceived rewards at chance level, only the frequency but not the peak velocity of saccades was enhanced by rewards. The latter observation hinted toward the possibility that truly subliminal rewards solely enhance the saccade frequency whereas the saccade peak velocity is only enhanced at higher levels of visibility. To test this possibility, we performed a second control experiment.

### Control Experiment: Reproducibility of Results in an Independent Sample of Participants with a Strict Control of Subliminal Perception of Reward Incentives

**Figure 5.**
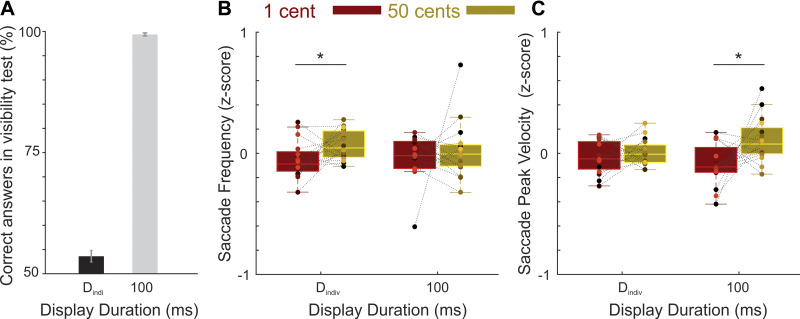
Influence of reward incentives on saccadic parameters in the control experiment. *A*. the subjective visibility of coin images at each display duration. *B*: the effect of low (1 cent, copper color) and high (50 cents, gold color) reward incentives on the saccade frequency. *C*: same as *B* for the saccadic peak velocity. Colored dots depict the data of individual participants. *P < 0.05.

In the control experiment, a long (*T* = 100 ms) and a short display duration (*T* = D_indiv_) were tested (Fig. 5*A*). By adjusting the luminance contrast of the coin images in addition to their display duration, we ensured that at D_indiv_ all participants reached a subliminal level of perception (see also materials and methods and Supplemental Materials). In fact, the analysis of the 4AFC visibility test confirmed that both criteria of subliminal perception were met at D_indiv_, as the correct responses were at chance level (means ± SD = 52.34 ± 5.28%, *P* = 0.34, one-tailed, one-sample *t* test) and the probability of seen responses was not significantly different from zero (means ± SD = 1.48 ± 2.82%, *P* = 0.31, one-tailed, one-sample *t* test). Binomial tests on the data of individual subjects, confirmed that all participants had reached a subliminal level of perception at D_indiv_ (binomial test *P* > 0.05). At 100 ms, correct responses were significantly higher than the chance level (means ± SD = 99.21 ± 1.5%, *P* < 10^−14^, one-tailed, one-sample *t* test for a difference from chance level, i.e., 50%) suggesting that participants perceived the reward incentives consciously.

As the main aim of the control experiment was to test the possibility of a dissociation between motivational effects of subliminal rewards on saccades’ frequency and velocity, we focused on these saccadic parameters (Fig. 5). To this end, the *z*-scored frequency or velocity of the saccades were subjected to a repeated-measures ANOVA (with 3 within-subjects factors: reward, display duration, and the saccade parameter: frequency vs. velocity). This analysis revealed a significant main effect of reward [*F*(1,15) = 5.78, *P* = 0.03, $\eta_{p}^{2}$= 0.28] and a trend for a three-way reward × display duration × saccade parameter interaction [*F*(1,15) = 3.37, *P* = 0.086, $\eta_{p}^{2}$= 0.18]. Post hoc pairwise comparisons showed that the effect of reward on saccades’ frequency was only significant at D_indiv_ where perception was subliminal (for *T* = D_indiv_: *P* = 0.039 and *d*_z_ = 0.57; for *T* = 100 ms: *P* = 0.567 and *d*_z_ = 0.15). Analysis of the saccadic peak velocity revealed an opposite pattern, as the effect of reward was only significant at 100 ms and not at D_indiv_ (for *T* = D_indiv_: *P* = 0.401 and *d*_z_ = 0.22; for *T* = 100 ms: *P* = 0.024; and *d*_z_ = 0.63). Other main and interaction effects did not reach significance [*F*(1,15) <0.1, *P* > 0.1].

Therefore, in the control experiment the effect of subliminal rewards on saccade frequency but not peak velocity was replicated. The three-way reward × display duration × saccade parameter interaction however, did not reach statistical significance suggesting that whereas the saccade frequency may be a more sensitive indicator of subliminal motivational effects, there is no functional dissociation between the effects of reward on the modulation of saccadic frequency and peak velocity.

Finally, we ruled out that our results are due to differences in the noise level of eye position data across different individuals and experimental conditions. To this end, we inspected our results when saccades were detected based on an adaptive algorithm introduced by Nyström and Holmqvist (35). This method can be applied to the data of individual trials of each subject, therefore adjusting for different levels of noise across participants and conditions. Using this analysis, we found similar results to the ones reported thus far in both experiments (for details see the Supplemental Materials) demonstrating that our findings are independent of the method used to detect the saccades.

## DISCUSSION

Previous studies have shown that reward incentives enhance the exerted effort in prospect of a higher reward, even when presented subliminally (6, 7, 37). The aim of the current study was to test whether these effects also extend to eye movements. In our first experiment, we observed an enhancement of all measures of oculomotor effort; including the number of hits, frequency and peak velocity of all saccades; when higher reward was at stake and reward cues were shown long enough to be consciously perceived. The reward-driven enhancement of oculomotor effort also occurred at the shortest display duration when reward cues were perceived subliminally. However, when a more stringent criterion at the individual level was used to determine the subliminal perception, only the frequency but not the peak velocity of saccades was found to be significantly modulated by rewards. This pattern was also replicated in a second control experiment where the stimulus presentation was strictly controlled to yield subliminal perception in every individual. Although at the subliminal threshold a larger effect size was found for the reward-driven modulation of the saccade frequency compared to the peak velocity, this difference did not reach statistical significance when both parameters were tested in the same statistical model. These results therefore demonstrate a robust effect of reward incentives on multiple aspects of goal-directed eye movements that can also extend to situations when rewards are perceived subliminally. Moreover, our results indicate that the frequency of saccades may be the most sensitive measure of subliminal incentivization of eye movements.

The results obtained with the consciously perceived reward cues are in line with previous studies showing a modulatory effect of supraliminal reward incentives on saccade metrics (17, 20, 23, 38). This finding further indicates that humans can voluntarily adjust their oculomotor performance, through changing the frequency and velocity of their eye movements, depending on the magnitude of expected rewards. As such, these results confirm previous findings that saccade metrics could be, at least to some extent, voluntarily controlled (20, 24). Importantly, the invigorating effect of rewards also occurred when the stereotypical relationship between the saccade amplitude and velocity was factored out, as shown by the within-subjects measure of saccade vigor. We note, however, that the energizing effect of rewards on the saccade metrics could also be driven by involuntary mechanisms, as rewards have been shown to exert automatic/reflexive modulation of saccades (39). The conjoint involvement of voluntary and involuntary mechanisms is supported by theoretical frameworks suggesting two distinct influences of reward motivation on behavior (2, 40). According to these, goal-directed effects determine the current goal of behavior and affect behavior in an “outcome-specific” manner. In contrast, “energizing” effects of reward are more general and determine the vigor of all actions in an “outcome-unspecific: and involuntary manner (2, 40). As such the motivational effects of reward in the current study may be a combination of both voluntary and involuntary mechanisms.

Whereas consciously perceived reward cues enhanced all measures of oculomotor effort, at a subliminal threshold the effect sizes were smaller, and for some measures of performance, such as the hit rates and saccadic vigor, did not reach statistical significance. Moreover, in participants who had reached a subliminal level of perception at the shortest display duration, only the frequency but not the peak velocity of saccades was significantly modulated by rewards. However, in both experiments reported here, we found only a trend for a significant dissociation between the reward-driven modulation of the saccade frequency and peak velocity when both measures were assessed in the same statistical models. Therefore, our results overall demonstrate that the effortful planning and execution of rapid eye movements can be incentivized by subliminal rewards, although with different strengths across different metrics of the saccades. Although we did not perform neurophysiological measurements in this study, our findings can be understood and expanded in light of previous studies. These studies have demonstrated that the burst activity of neurons in Superior Colliculus (SC) underlies saccade initiation (41, 42) and SC neurons are also involved in the adjustment of saccades’ peak velocity (43–45). Collicular neurons receive inputs from a large number of cortical and subcortical structures involved in the processing of reward information (for reviews see Refs 15 and 46). One possibility is that subliminal rewards can elicit a sufficient activation of the subcortical structures involved in the reward-driven modulation of SC neurons, thereby enhancing the overall excitability of the saccade generating neurons and hence the frequency of the saccades. An additional mechanism however may be needed to control the saccade peak velocity once eye movements toward a target are initiated, so that the maximum velocity is adjusted both based on the spatial position of the saccade target and the level of incentives. This latter mechanism may need a finer, moment-to-moment adjustment of saccade parameters and perhaps a larger involvement of the supervisory cortical network involved in the goal-directed planning of eye movements that is achieved at a higher level of conscious awareness. Such a mechanism can explain the larger effect sizes found for the effect of subliminal rewards on the saccade frequency compared to the peak velocity/vigor, which is in line with previous studies showing differences between the motivational enhancement of saccadic speed versus vigor (47, 48), but also entails that at a certain level of conscious awareness of reward incentives both saccadic parameters may be simultaneously influenced. The behavioral paradigm devised here can be employed in future neurophysiological and neuroimaging studies to unravel the balance between subcortical and cortical mechanisms involved in the subliminal motivational modulation of eye movements.

Future research is also needed to elucidate whether the pattern of results we obtained is a general characteristic of motivational effects on eye movements or rather depend on the specific features of the task we employed. Since incentive-dependent adjustment of exerted effort critically depends on task difficulty (28–30), and execution of eye movements is not as demanding as the tasks previously used to investigate the subliminal reward effects (7, 10, 12, 13), we employed a novel paradigm that was specifically designed to be maximally taxing for the eye movements. One aspect of the task we used is particularly important in this respect: participants needed to plan a sequence of eye movements back and forth between a fixation point and several peripheral targets. In a previous study, it was shown that when humans are required to execute saccades to a large number of target locations, saccade preparation for all target locations is carried out in parallel (49). It is possible that at a subliminal threshold the way a sequence of saccades is planned is different from how saccades are planned at higher visibility levels. In fact, the strategy used for planning saccades to a sequence of targets may discourage careful planning of individual eye movements in favor of increasing the frequency compared with boosting the velocity and/or accuracy of the saccades, as has been shown previously (50). This can account for a larger effect of subliminal rewards on the saccade frequency compared to peak velocity at the subliminal threshold. In addition, participants received immediate feedback after a saccade had landed its intended destination, i.e., either the fixation point or one of the peripheral targets. These features indicate that in addition to adjusting the velocity/vigor of the saccades, participants needed to exert some degree of cognitive effort to keep track of the sequence of the eye movements and the feedback information. Since we did not test a condition in which successful realization of the task only required motoric but not cognitive effort, we cannot isolate the individual contribution of these factors to our effects.

Although previous studies have investigated how different levels of conscious awareness of saccade goals affect the eye movements, our study is the first to investigate the subconscious incentivization of saccade metrics. In one previous study (51), it was shown that participants’ saccadic performance was completely unrelated to their awareness of the saccade goal. A more recent study showed that participants have a gaze bias toward a stimulus that they are completely unaware of its position and identity (52). Finally, in a free viewing task, it was shown that visual cues presented below the awareness threshold could influence the direction and latency of the saccades (53). Based on these findings, it has been suggested that saccadic selection is primarily driven by subconscious processes. This idea is further supported by a more general theoretical framework proposing a dissociation between action and perception (54), accounting for cases where participants can be unaware of the perceptual features of a saccade target, but could nevertheless execute flawless actions toward them. Our results are in general agreement with these previous findings, as we provide evidence for the modulation of eye movements by subliminal factors. In addition, by examining the influence of subliminal rewards on multiple metrics of saccadic eye movements, our study inspires a closer examination of the oculomotor planning to reveal similarities and differences in how different aspects of oculomotor responses are influenced by subliminal factors.

Although a wealth of previous research has demonstrated that goal-directed behavior could be controlled by subconscious processes, a great deal of controversy surrounds the reported effects (for a review see Ref. 55). One important concern regarding subliminal effects is the uncertainty regarding whether participants had truly reached a subconscious threshold (56). Typically, researchers try to overcome this difficulty by showing that subliminal cues are not perceived above chance at a group level. However, this method does not consider the cases when a group consists of a mix between individuals who had reached the subliminal threshold and those who had not, as was also the case in our main experiment. To overcome this problem, we applied two strategies. First, to account for the different levels of conscious awareness of reward at the shortest display duration across participants, we analyzed our data while including each individual’s visibility score as a covariate. Although this method adjusts our estimated effect sizes for the degree to which participants were able to discriminate the reward magnitude, it does not ensure that our effects are not primarily driven by participants who perceived reward cues supraliminally. To account for this, we used a strict measure for subliminal perception at the individual level to decide whether each participant had reached subliminal perception or not in the main experiment, and subsequently adjusted our stimuli to ensure subliminal perception of reward in all individuals participating in the control experiment. Using these measures, we were able to detect the saccadic parameters (i.e., the saccade frequency) that exhibit a robust effect of reward incentives when the variability of subjective experience of rewards across participants was controlled. We further note that the perception of reward may fluctuate within the same individual and task condition so that in some trials reward cues are perceived consciously and, in some other trials, subconsciously. To detect such cases, one could measure the level of conscious awareness on a trial-by-trial basis, rather than ascribing all trials of a certain condition to a fixed sub- or supraliminal category. We decided against inquiring participants about their subjective experience on a trial-by-trial basis to avoid a potential perceptual or postperceptual bias introduced by the overt reports on participants’ performance in the eye movement task and used instead an independent task to determine the visibility of reward cues at each duration. A possibility to consider for future studies would be to use no-report paradigms (57) to assess the conscious awareness of reward cues, thus minimizing biases induced by overt reports while allowing a trial-by-trial assessment of the level of conscious awareness.

Taken together, our results suggest that multiple aspects of reward-driven oculomotor planning could be modulated by subconscious processes. Our study underscores the importance of understanding oculomotor planning as a means to gain insight into underlying mechanisms of cognition. Saccades play a pivotal role in acquiring sensory information across space and are closely linked to attentional selection. Previous studies have shown that saccade metrics could be used to probe different aspects of decision making in both perceptual (58) as well as value-based decision making (22). Our study furthers these previous findings and additionally shows that saccades provide detailed and precise information regarding how one of the most complex aspects of human cognition, namely that of conscious awareness, is orchestrated.

## SUPPLEMENTAL DATA

Supplemental Material: https://doi.org/10.17605/OSF.IO/BFSZW.

## GRANTS

This work was initially supported by a seed fund grant from Leibniz ScienceCampus Primate Cognition, Göttingen, Germany to M.W. and A.P. and continued by an ERC Starting Grant (716846) to A.P.

## DISCLOSURES

No conflicts of interest, financial or otherwise, are declared by the authors.

## AUTHOR CONTRIBUTIONS

V.K.H., O.U., M.W., and A.P. conceived and designed research; V.K.H., O.U., and J.E.A. performed experiments; V.K.H., O.U., J.E.A., and A.P. analyzed data; V.K.H., O.U., M.W., and A.P. interpreted results of experiments; V.K.H., O.U., and A.P. prepared figures; V.K.H. and A.P. drafted manuscript; V.K.H., O.U., J.E.A., M.W., and A.P. edited and revised manuscript; V.K.H., O.U., J.E.A., M.W., and A.P. approved final version of manuscript.

## ENDNOTE

At the request of the authors, readers are herein alerted to the fact that additional materials related to this manuscript may be found at https://osf.io/bfszw/. These materials are not a part of this manuscript and have not undergone peer review by the American Physiological Society (APS). APS and the journal editors take no responsibility for these materials, for the website address, or for any links to or from it.

## References

[1] Pessoa L, Engelmann JB. Embedding reward signals into perception and cognition. Front Neurosci 4: 17, 2010. doi:10.3389/fnins.2010.00017. 20859524 PMC2940450

[2] Dickinson A, Balleine B. Motivational control of goal-directed action. Animal Learning & Behavior 22: 1–18, 1994. doi:10.3758/BF03199951.

[3] Bargh JA, Gollwitzer PM, Lee-Chai A, Barndollar K, Trötschel R. The automated will: nonconscious activation and pursuit of behavioral goals. J Pers Soc Psychol 81: 1014–1027, 2001. 11761304 PMC3005626

[4] Hart W, Albarracín D. The effects of chronic achievement motivation and achievement primes on the activation of achievement and fun goals. J Pers Soc Psychol 97: 1129–1141, 2009. doi:10.1037/a0017146. 19968423 PMC3626449

[5] Custers R, Aarts H. Positive affect as implicit motivator: on the nonconscious operation of behavioral goals. J Pers Soc Psychol 89: 129–142, 2005. doi:10.1037/0022-3514.89.2.129. 16162049

[6] Bijleveld E, Custers R, Aarts H. The unconscious eye opener: pupil dilation reveals strategic recruitment of resources upon presentation of subliminal reward cues. Psychol Sci 20: 1313–1315, 2009. doi:10.1111/j.1467-9280.2009.02443.x. 19788532

[7] Pessiglione M, Schmidt L, Draganski B, Kalisch R, Lau H, Dolan RJ, Frith CD. How the brain translates money into force: a neuroimaging study of subliminal motivation. Science 316: 904–906, 2007. doi:10.1126/science.1140459. 17431137 PMC2631941

[8] Bijleveld E, Custers R, Aarts H. Unconscious reward cues increase invested effort, but do not change speed-accuracy tradeoffs. Cognition 115: 330–335, 2010. doi:10.1016/j.cognition.2009.12.012. 20089247

[9] Bijleveld E, Custers R, Aarts H. Human reward pursuit: from rudimentary to higher-level functions. Curr Dir Psychol Sci 21: 194–199, 2012. doi:10.1177/0963721412438463.

[10] Capa RL, Bustin GM, Cleeremans A, Hansenne M. conscious and unconscious reward cues can affect a critical component of executive control. Exp Psychol 58: 370–375, 2011. doi:10.1027/1618-3169/a000104. 21310696

[11] Bijleveld E, Custers R, Van der Stigchel S, Aarts H, Pas P, Vink M. Distinct neural responses to conscious versus unconscious monetary reward cues. Hum Brain Mapp 35: 5578–5586, 2014. doi:10.1002/hbm.22571. 24984961 PMC4265283

[12] Zedelius CM, Veling H, Aarts H. When unconscious rewards boost cognitive task performance inefficiently: the role of consciousness in integrating value and attainability information. Front Hum Neurosci 6: 219, 2012. doi:10.3389/fnhum.2012.00219. 22848198 PMC3404454

[13] Bijleveld E, Custers R, Aarts H. Adaptive reward pursuit: how effort requirements affect unconscious reward responses and conscious reward decisions. J Exp Psychol Gen 141: 728–742, 2012. doi:10.1037/a0027615. 22468672

[14] Trommershäuser J, Glimcher PW, Gegenfurtner KR. Visual processing, learning and feedback in the primate eye movement system. Trends Neurosci 32: 583–590, 2009. doi:10.1016/j.tins.2009.07.004. 19729211

[15] Hikosaka O. Basal ganglia mechanisms of reward-oriented eye movement. Ann NY Acad Sci 1104: 229–249, 2007. doi:10.1196/annals.1390.012. 17360800

[16] Itoh H, Nakahara H, Hikosaka O, Kawagoe R, Takikawa Y, Aihara K. Correlation of primate caudate neural activity and saccade parameters in reward-oriented behavior. J Neurophysiol 89: 1774–1783, 2003. doi:10.1152/jn.00630.2002. 12686566

[17] Takikawa Y, Kawagoe R, Itoh H, Nakahara H, Hikosaka O. Modulation of saccadic eye movements by predicted reward outcome. Exp Brain Res 142: 284–291, 2002. doi:10.1007/s00221-001-0928-1. 11807582

[18] Chen LL, Hung LY, Quinet J, Kosek K. Cognitive regulation of saccadic velocity by reward prospect. Eur J Neurosci 38: 2434–2444, 2013. doi:10.1111/ejn.12247. 23668781

[19] Bahill AT, Clark MR, Stark L. The main sequence, a tool for studying human eye movements. Mathematical Biosciences 24: 191–204, 1975. doi:10.1016/0025-5564(75)90075-9.

[20] Chen LL, Chen YM, Zhou W, Mustain WD. Monetary reward speeds up voluntary saccades. Front Integr Neurosci 8: 48, 2014. doi:10.3389/fnint.2014.00048 24994970 PMC4064668

[21] Reppert TR, Lempert KM, Glimcher PW, Shadmehr R. Modulation of saccade vigor during value-based decision making. J Neurosci 35: 15369–15378, 2015. doi:10.1523/JNEUROSCI.2621-15.2015. 26586823 PMC4649007

[22] Shadmehr R, Reppert TR, Summerside EM, Yoon T, Ahmed AA. Movement vigor as a reflection of subjective economic utility. Trends Neurosci 42: 323–336, 2019. doi:10.1016/j.tins.2019.02.003. 30878152 PMC6486867

[23] Choi JES, Vaswani PA, Shadmehr R. Vigor of movements and the cost of time in decision making. J Neurosci 34: 1212–1223, 2014. doi:10.1523/JNEUROSCI.2798-13.2014. 24453313 PMC3898284

[24] Muhammed K, Dalmaijer E, Manohar S, Husain M. Voluntary modulation of saccadic peak velocity associated with individual differences in motivation. Cortex 122: 198–212, 2020. doi:10.1016/j.cortex.2018.12.001. 30638586 PMC6970223

[25] Schall JD. Production, control, and visual guidance of saccadic eye movements. ISRN Neurol 2013: 752384, 2013. doi:10.1155/2013/752384. 24260720 PMC3821953

[26] Munoz DP. Commentary: saccadic eye movements: overview of neural circuitry. Prog Brain Res 140: 89–96, 2002. doi:10.1016/S0079-6123(02)40044-1. 12508584

[27] Ding L, Hikosaka O. Comparison of reward modulation in the frontal eye field and caudate of the macaque. J Neurosci 26: 6695–6703, 2006. doi:10.1523/JNEUROSCI.0836-06.2006. 16793877 PMC6673837

[28] Brehm JW, Self EA. The intensity of motivation. Annu Rev Psychol 40: 109–131, 1989. doi:10.1146/annurev.ps.40.020189.000545. 2648973

[29] Capa RL. Clarifying achievement motives and effort: studies of cardiovascular response. In: How Motivation Affects Cardiovascular Response: Mechanisms and Applications, edited by Wright RA, Gendolla GHE. Washington, DC: American Psychological Association, 2011, p. 383–398. doi:10.1037/13090-019.

[30] Gendolla GHE, Richter M. Effort mobilization when the self is involved: some lessons from the cardiovascular system. Rev Gen Psychol 14: 212–226, 2010. doi:10.1037/a0019742.

[31] Shadmehr R, Ahmed AA. Vigor: Neuroeconomics of Movement Control (Online). Cambridge, MA: MIT Press, 2020. doi:10.7551/mitpress/12940.001.0001. 33261698

[32] Faul F, Erdfelder E, Lang AG, Buchner A. G*Power 3: a flexible statistical power analysis program for the social, behavioral, and biomedical sciences. Behav Res Methods 39: 175–191, 2007. doi:10.3758/bf03193146. 17695343

[33] Brainard DH. The Psychophysics Toolbox. Spat Vis 10: 433–436, 1997. doi:10.1163/156856897X00357. 9176952

[34] Cornelissen FW, Peters EM, Palmer J. The EyeLink toolbox: eye tracking with MATLAB and the Psychophysics Toolbox. Behav Res Methods Instrum Comput 34: 613–617, 2002. doi:10.3758/BF03195489. 12564564

[35] Nyström M, Holmqvist K. An adaptive algorithm for fixation, saccade, and glissade detection in eyetracking data. Behav Res Methods 42: 188–204, 2010. doi:10.3758/BRM.42.1.188. 20160299

[36] Lakens D. Calculating and reporting effect sizes to facilitate cumulative science: a practical primer for t-tests and ANOVAs. Front Psychol 4: 863, 2013. doi:10.3389/fpsyg.2013.00863. 24324449 PMC3840331

[37] Zedelius CM, Veling H, Custers R, Bijleveld E, Chiew KS, Aarts H. A new perspective on human reward research: how consciously and unconsciously perceived reward information influences performance. Cogn Affect Behav Neurosci 14: 493–508, 2014. doi:10.3758/s13415-013-0241-z. 24399682

[38] Hikosaka O, Takikawa Y, Kawagoe R. Role of the basal ganglia in the control of purposive saccadic eye movements. Physiol Rev 80: 953–978, 2000. doi:10.1152/physrev.2000.80.3.953. 10893428

[39] Manohar SG, Finzi RD, Drew D, Husain M. Distinct motivational effects of contingent and noncontingent rewards. Psychol Sci 28: 1016–1026, 2017. doi:10.1177/0956797617693326. 28488927 PMC5510684

[40] Niv Y, Joel D, Dayan P. A normative perspective on motivation. Trends Cogn Sci 10: 375–381, 2006. doi:10.1016/j.tics.2006.06.010. 16843041

[41] Wurtz RH, Goldberg ME. Superior colliculus cell responses related to eye movements in awake monkeys. Science 171: 82–84, 1971. 10.1126/science.171.3966.82. 4992313

[42] Sparks DL. Functional properties of neurons in the monkey superior colliculus: coupling of neuronal activity and saccade onset. Brain Res 156: 1–16, 1978. doi:10.1016/0006-8993(78)90075-6. 100173

[43] Stanford TR, Freedman EG, Sparks DL. Site and parameters of microstimulation: evidence for independent effects on the properties of saccades evoked from the primate superior colliculus. J Neurophysiol 76: 3360–3381, 1996. doi:10.1152/jn.1996.76.5.3360. 8930279

[44] Katnani HA, Gandhi NJ. The relative impact of microstimulation parameters on movement generation. J Neurophysiol 108: 528–538, 2012. doi:10.1152/jn.00257.2012. 22539818 PMC3404793

[45] Lee C, Rohrer WH, Sparks DL. Population coding of saccadic eye movements by neurons in the superior colliculus. Nature 332: 357–360, 1988. doi:10.1038/332357a0. 3352733

[46] Hikosaka O, Kim HF, Yasuda M, Yamamoto S. Basal ganglia circuits for reward value–guided behavior. Annu Rev Neurosci 37: 289–306, 2014. doi:10.1146/annurev-neuro-071013-013924. 25032497 PMC4148825

[47] Manohar SG, Chong TT-J, Apps MAJ, Batla A, Stamelou M, Jarman PR, Bhatia KP, Husain M. Reward pays the cost of noise reduction in motor and cognitive control. Curr Biol 25: 1707–1716, 2015. doi:10.1016/j.cub.2015.05.038. 26096975 PMC4557747

[48] Grogan JP, Sandhu TR, Hu MT, Manohar SG. Dopamine promotes instrumental motivation, but reduces reward-related vigour. eLife 9: e58321, 2020. doi:10.7554/eLife.58321. 33001026 PMC7599069

[49] McSorley E, Gilchrist ID, McCloy R. The programming of sequences of saccades. Exp Brain Res 237: 1009–1018, 2019. doi:10.1007/s00221-019-05481-7. 30725153 PMC6430760

[50] Wu CC, Kwon OS, Kowler E. Fitts’s Law and speed/accuracy trade-offs during sequences of saccades: Implications for strategies of saccadic planning. Vision Res 50: 2142–2157, 2010. doi:10.1016/j.visres.2010.08.008. 20709093 PMC2949070

[51] van Zoest W, Donk M. Awareness of the saccade goal in oculomotor selection: your eyes go before you know. Conscious Cogn 19: 861–871, 2010. doi:10.1016/j.concog.2010.04.001. 20418114

[52] Rothkirch M, Stein T, Sekutowicz M, Sterzer P. A direct oculomotor correlate of unconscious visual processing. Curr Biol 22: R514–R515, 2012. doi:10.1016/j.cub.2012.04.046. 22789995

[53] Huang YF, Tan EGF, Soon CS, Hsieh PJ. Unconscious cues bias first saccades in a free-saccade task. Conscious Cogn 29: 48–55, 2014. doi:10.1016/j.concog.2014.07.009. 25108793

[54] Milner AD, Goodale MA. Two visual systems re-viewed. Neuropsychologia 46: 774–785, 2008. doi:10.1016/j.neuropsychologia.2007.10.005. 18037456

[55] Newell BR, Shanks DR. Unconscious influences on decision making: a critical review. Behav Brain Sci 37: 1–19, 2014. doi:10.1017/S0140525X12003214. 24461214

[56] Vadillo MA, Konstantinidis E, Shanks DR. Underpowered samples, false negatives, and unconscious learning. Psychon Bull Rev 23: 87–102, 2016. doi:10.3758/s13423-015-0892-6. 26122896 PMC4742512

[57] Tsuchiya N, Wilke M, Frässle S, Lamme VAF. No-report paradigms: extracting the true neural correlates of consciousness. Trends Cogn Sci 19: 757–770, 2015. doi:10.1016/j.tics.2015.10.002. 26585549

[58] Seideman JA, Stanford TR, Salinas E. Saccade metrics reflect decision-making dynamics during urgent choices. Nat Commun 9: 2907, 2018. doi:10.1038/s41467-018-05319-w. 30046066 PMC6060154

